# Generation of comprehensive transposon insertion mutant library for the model archaeon, *Haloferax volcanii*, and its use for gene discovery

**DOI:** 10.1186/s12915-014-0103-3

**Published:** 2014-12-09

**Authors:** Saija Kiljunen, Maria I Pajunen, Kieran Dilks, Stefanie Storf, Mechthild Pohlschroder, Harri Savilahti

**Affiliations:** Division of Genetics and Physiology, Department of Biology, University of Turku, Turku, Finland; Department of Biology, University of Pennsylvania, Philadelphia, PA USA; Current address: Department of Biosciences, Division of Biochemistry and Biotechnology, University of Helsinki, Helsinki, Finland

**Keywords:** *Haloferax volcanii*, Halophilic archaea, Insertion mutant library, Mu transposition, MuA protein, Gene discovery

## Abstract

**Background:**

Archaea share fundamental properties with bacteria and eukaryotes. Yet, they also possess unique attributes, which largely remain poorly characterized. *Haloferax volcanii* is an aerobic, moderately halophilic archaeon that can be grown in defined media. It serves as an excellent archaeal model organism to study the molecular mechanisms of biological processes and cellular responses to changes in the environment. Studies on haloarchaea have been impeded by the lack of efficient genetic screens that would facilitate the identification of protein functions and respective metabolic pathways.

**Results:**

Here, we devised an insertion mutagenesis strategy that combined Mu *in vitro* DNA transposition and homologous-recombination-based gene targeting in *H. volcanii*. We generated an insertion mutant library, in which the clones contained a single genomic insertion. From the library, we isolated pigmentation-defective and auxotrophic mutants, and the respective insertions pinpointed a number of genes previously known to be involved in carotenoid and amino acid biosynthesis pathways, thus validating the performance of the methodologies used. We also identified mutants that had a transposon insertion in a gene encoding a protein of unknown or putative function, demonstrating that novel roles for non-annotated genes could be assigned.

**Conclusions:**

We have generated, for the first time, a random genomic insertion mutant library for a halophilic archaeon and used it for efficient gene discovery. The library will facilitate the identification of non-essential genes behind any specific biochemical pathway. It represents a significant step towards achieving a more complete understanding of the unique characteristics of halophilic archaea.

**Electronic supplementary material:**

The online version of this article (doi:10.1186/s12915-014-0103-3) contains supplementary material, which is available to authorized users.

## Background

According to the three domains of life paradigm, archaea are distinct life forms and constitute a domain of their own, the other domains being bacteria and eukarya [1,2]. Recent studies suggest that eukaryotes may, in fact, have originated from ancient archaea [3,4]. Many structural and functional attributes of archaea share a high degree of similarity with the corresponding features present in bacterial or eukaryotic cells. Yet, several aspects of archaeal biochemistry and biology are unique [5]. These include, for example, the ether linkages between glycerol and hydrophobic side chains in archaeal membrane phospholipids [6,7] and the archaeal cell wall, often composed of a single S-layer glycoprotein [8,9]. Moreover, the ability of many archaea to thrive in extreme environments is biologically intriguing and makes archaea a valuable resource for biotechnology applications. Even though a degree of information is available about some unique archaeal features, the biochemical pathways and genetic basis behind many of them remain poorly characterized or entirely uncharacterized.

*Haloferax volcanii* is a moderately halophilic, mesophilic and aerobic archaeon that can be cultured in defined media, and it serves as an excellent model organism for the archaeal domain of life. The genome consists of one major chromosome (2.8 Mb), present in up to thirty copies, and three secondary minichromosomes pHV1 (85 kb), pHV3 (438 kb), and pHV4 (636 kb), with copy numbers up to 21, 26, and 17, respectively [10,11]. In addition, the characterized wild type strains also harbor a plasmid pHV2 (6.3 kb) [10]. A number of biochemical, genetic and molecular biology methods have been developed for *H. volcanii* [12,13], and their successful employment has enabled the identification of key factors involved in several crucial cellular functions, including the components of the two predominant protein transport systems [14,15] and the proteasome [16]. While several types of useable genome manipulation techniques and selectable markers are available for *H. volcanii* [13], its more detailed biological characterization remains critically dependent on the development of a wider selection of advanced methodologies.

Biochemical pathways of archaea can be discovered using comparative genome analyses, where homologous bacterial or eukaryotic genes may indicate a functional archaeal counterpart. However, these types of approaches have only a limited utility in identifying genes behind unique archaeal features. Some archaeal genes may be identified through biochemical means, but the low expression level of many gene products often complicates these analyses. In general, genetics provides the most effective means to discover novel genes, but sophisticated genetics methodologies are yet to be developed for most archaeal species.

A comprehensive collection of mutant clones with randomly distributed genomic alterations provides a valuable resource for studies aimed at delineating molecular mechanisms behind biological functions. Such libraries have proven their immense usefulness, particularly in microbiological research, but only a limited number of archaeal studies have exploited such resources. Chemical and physical mutagens, such as ethyl methanesulfonate (EMS) and ultraviolet light, have been used to introduce random mutations into several archaeal species [17,18]. Although these studies have yielded auxotrophic mutants, mapping of the underlying genetic alterations has been an arduous task. Furthermore, the presence of multiple genome copies within a single cell [11], a common feature of archaea, complicates these types of approaches: mutagenesis produces heterozygous cells with initially only one copy of a mutated allele, and without a selective pressure, newly acquired mutations may be lost or fail to result in detectable phenotypic changes due the dominance of the wild type alleles [19].

Insertional mutagenesis typically employs selectable markers to tag random genomic loci, and transposition-based methods offer the most straightforward general means for the purpose. Thus far, genome-wide transposon insertion libraries have only been produced for two archaeal species, anaerobic methanogens *Methanosarcina acetivorans* [20] and *Methanococcus maripaludis* [21]. Some progress in transposon mutagenesis has also been made with halophilic *Haloarcula hispanica* [22,23], but comprehensive insertion mutant libraries have not been produced for any haloarchaeal species.

All transposon-mediated mutagenesis approaches employ efficient transpositional recombination machinery, which catalyzes the joining of the transposon DNA into target DNA, and the most useful transposition systems integrate into a nearly random selection of target sites [24]. The machinery is normally introduced into target cells by the use of plasmid vehicles [25] or via electroporation of pre-assembled DNA transposition complexes, transpososomes [26,27]. The plasmid vehicle strategy requires a functional transposition system, either native or of heterologous origin. However, no native transposon has so far been recruited for mutagenesis in *H. volcanii*, and heterologous systems may not be functional due to the high (1 to 2 M) intracellular potassium chloride concentration in haloarchaea. The transpososome delivery strategy is critically dependent on electrotransformation under salt-free conditions, and as *H. volcanii* cells are sensitive to a low salt concentration, the utilization of such a strategy may be difficult or impossible. However, alternative approaches may be used, if the organism harbors homologous recombination machinery sufficiently operational for efficient gene targeting. In principle, insertion mutant libraries can be generated by the combined use of *in vitro* transposition mutagenesis and simultaneous targeting of a mixture of genomic fragments, as shown with yeast and fungi [28,29].

Previous studies have shown that the *H. volcanii* homologous recombination machinery functions for gene targeting [30-32], and that the MuA-catalyzed *in vitro* DNA transposition reaction can be used to introduce randomly distributed insertions into any target DNA [33]. Encouraged by these achievements, we developed here a strategy to generate an insertion mutant library for *H. volcanii* and generated a broad collection of clones, each individually tagged with transposon DNA. We validated the quality of the library for gene discovery by isolating pigmentation-defective and auxotrophic mutants. The library represents the first comprehensive insertion mutant collection generated in haloarchaea. As the applied strategy is characteristically species non-specific, it should readily be transferable to other archaeal species.

## Results

### Plasmid library with *H. volcanii* inserts tagged with transposon DNA

**Figure 1:**
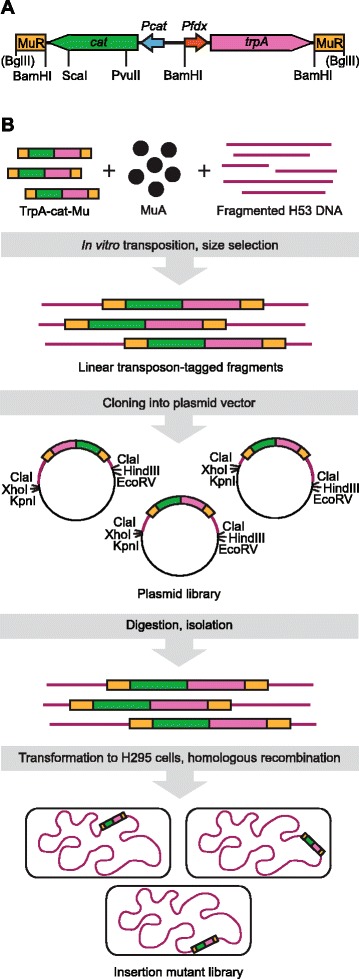
**Transposon TrpA-cat-Mu and overview of the transposon mutagenesis strategy. (A)** The transposon was made from Cat-Mu [33] by the addition of a gene cassette containing *trpA* and *Pfdx*. Orange rectangles indicate 50 bp of Mu R-end DNA. The *trpA* gene (pink arrow) under the control of *Pfdx* promoter (red arrow) was used for the selection in *H. volcanii*; the *cat* gene (green arrow) under the *cat* promoter (blue arrow) was used for the selection in *E. coli*. The transposon was released from its carrier plasmid by BglII digestion. **(B)** *H. volcanii* H53 DNA was partially digested with HpaII, AciI, and TaqI and used as a target in an *in vitro* Mu transposition reaction with TrpA-cat-Mu as a donor DNA. From transposition products, 4 to 6 kb fragments were isolated and cloned into the ClaI site of pBlueSript SK+ to yield a plasmid library. Inserts were released by digesting with XhoI + HindIII or KpnI + EcoRV, gel-purified, and used to transform *H. volcanii* H295 cells to generate a transposon insertion mutant library.

Initially, we wanted to generate a plasmid library, which would contain transposon-tagged *H. volcanii* genomic DNA segments as inserts, altogether covering the entire genome as overlapping fragments (Figure 1). For the tagging we made a shuttle transposon, TrpA-cat-Mu, which includes two marker genes, *cat* and *trpA*, allowing selection in *Escherichia coli* and *H. volcanii*, respectively (Figure 1A). Initially, *H. volcanii* genomic DNA was partially digested, in separate reactions, with three restriction enzymes (HpaII, AciI, TaqI), each recognizing a 4-bp sequence and generating a protruding 5′-GC. TrpA-cat-Mu was then integrated into the generated fragments using an *in vitro* transposition reaction, and the fragments were pooled. Following chromatographic size-selection, 4 to 6 kb fragments were cloned into a unique ClaI site of pSuperscript SK+, ultimately yielding a plasmid library with 28,000 independently generated member clones (Figure 1B). As *H. volcanii* harbors approximately 4,130 genes [10], on the average, each transposon-tagged gene should be present roughly seven times in this plasmid library.

To validate the plasmid library, we isolated plasmid DNA from three randomly selected clones. The *H. volcanii* DNA segment present in each of these plasmids (pSKT10, pSKT11, and pSKT12) was identified by sequencing. Plasmid pSKT10 harbored transposon-tagged DNA from the minor chromosome pHV3, whereas pSKT11 and pSKT12 contained tagged DNA from the main chromosome (Table 1). These data and the applied cloning strategy with strong biochemical and biological selections imply that each clone in the library includes transposon-tagged *H. volcanii* DNA.

**Table 1:** **Plasmids used in the work**

**Plasmid**	**Relevant feature**	**Reference/source**
pBlueScript SK+	*E. coli* cloning vector; Amp^r^	Stratagene
pUC19	*E. coli* cloning vector; Amp^r^	[34]
pTA231	*E. coli* – *H. volcanii* shuttle vector; Amp^r^, Trp^+^	[35]
pTA351	*E. coli* – *H. volcanii* shuttle vector; Amp^r^, Trp^+^	[36]
pMPH20	Carrier plasmid of transposon TrpA-cat-Mu; transposon released by BglII digestion. Amp^r^, Cam^r^, Trp^+^	This work
pSKT10	H295 pHV3 bases 228, 564 – 230, 899^a^ cloned in pBlueScript SK+. TrpA-cat-Mu inserted between HVO_B0199 and HVO_B0200. Amp^r^,Cam^r^, Trp^+^	This work
pSKT11	H295 chromosomal DNA bases 385, 714 – 390, 107^a^ cloned in pBlueScript SK+. TrpA-cat-Mu inserted in HVO_0434. Amp^r^, Cam^r^, Trp^+^	This work
pSKT12	H295 chromosomal DNA bases 1, 530, 448 – 1, 534, 708^a^ cloned in pBlueScript SK+. TrpA-cat-Mu inserted in HVO_1650. Amp^r^,Cam^r^, Trp^+^	This work

^a^Numbering according to *H. volcanii* D2 pHV3 (NC_013964.1) and genomic sequence (NC_013967.1), respectively. Amp: ampicillin; Cam: chloramphenicol.

### Validation of gene targeting procedure

Our initial targeting attempts with circular plasmid library DNA were problematic, yielding *H. volcanii* clones with no genomic transposon insertions but harboring replicative plasmids (data not shown, see Discussion). Therefore, we decided to use linear DNA fragments for targeting *en masse* (Figure 1B). Prior to the actual experiments, we wanted to validate the targeting procedure using known individual fragments. Accordingly, we isolated and gel-purified the inserts from the abovementioned plasmids, pSKT10, pSKT11, and pSKT12, using XhoI + HindIII double digestion. These fragments, named iSKT10, iSKT11, and iSKT12, were then used to transform *H. volcanii* H295 (Table 2) for gene targeting. This strain was chosen, as it portrays an elevated level of homologous recombination activity [37]. As dam methylation potentially influenced the outcome, we used both methylated and unmethylated DNA (isolated from *E. coli* strains DH5Δ and GM2929, respectively). The replicative plasmid pTA231 served as a control for the transformation of circular DNA (Table 3). While dam-methylation reduced the transformation efficiency of circular DNA by tenfold, the methylation status, surprisingly, did not influence the number of produced colonies in transformations with linear DNA. The efficiency of transformation varied among fragments, being 10^4^ to 10^5^ colony-forming units (CFU)/μg DNA with iSKT11 and iSKT12 but only 10^2^ CFU/μg DNA with iSKT10. These results likely directly reflect the differences in the length of the homology regions flanking the transposon DNA in these fragments (Table 3). The novel finding that methylation status had no effect on the transformation efficiency of linear fragments demonstrates that there is no need to passage the DNA to be transformed via a Dam^-^ bacterial strain. Accordingly, methylated DNA isolated from *E. coli* DH5α was used for further experiments.

**Table 2:** **Microbial strains used in the work**

**Strain**	**Genotype**	**Use**	**Reference/source**
*H. volcanii* H53	*pyrE2Δ trpA*Δ	Genomic DNA as target in *in vitro* mutagenesis	[35]
*H. volcanii* H295	*pyrE2Δ bgaHa-Kp trpAΔ mre11Δ rad50*Δ	Parental strain for library construction	[37]
*E. coli* DH10B	F^−^ *endA1 recA1 galE15 galK16 nupG rpsL ΔlacX74 80lacZΔM15 araD139 Δ(ara-leu)7697 mcrA (mrr-hsdRM-mrcBC)*	Construction of plasmid library	[38]
*E. coli* MC1061	F^–^ *araD139 Δ(ara-leu)7696 galE15 galK16 ΔlacX74 rpsL* (Str^r^) *hsdR2 (r*_*k*_^*−*^ *m*_*k*_^*+*^ *)mcrA mcrB1*	Standard cloning host	[39]
*E. coli* DH5α	F^−^ *endA1 supE*44 *thi-1 recA*1 *relA1 gyrA96 deoR nupG* 80*lacZΔM15 Δ(lacZYA-argF)U169 hsdR17 (r*_*k*_^*−*^ *m*_*k*_^*+*^ *)*	Isolation of methylated (Dam^+^) DNA	Invitrogen
*E. coli* GM2929	F^–^ *ara-14 leuB6 thi-1 fhuA31 lacY1 tsx-78 galK2 galT22 glnV44 hisG4 rpsL136* (Str^r^) *xyl-5 mtl-1 dam13::*Tn9 (Cam^r^) *dcm-6 mcrB1 hsdR2 (r*_*k*_^*−*^ *m*_*k*_^*+*^ *) mcrA recF143*	Isolation of unmethylated (Dam^-^) DNA	New England Biolabs

**Table 3:** **Efficiency of H295 transformation** ^**a,b**^

**DNA**	**Right flank (bp)**	**Left flank (bp)**	**Dam** ^**+**^	**Dam** ^**-**^
H_2_O	n.a. ^c^	n.a. ^c^	0	0
pTA231	n.a. ^c^	n.a. ^c^	1.3 × 10^4^	1.6 × 10^5^
iSKT10	2170	166	250	100
iSKT11	3074	1319	1.2 × 10^5^	1.1 × 10^5^
iSKT12	1326	2930	9.8 × 10^4^	2.3 × 10^4^

^a^Results from one representative experiment are shown; ^b^efficiency in CFU/μg DNA; ^c^n.a., not applicable.

**Figure 2:**
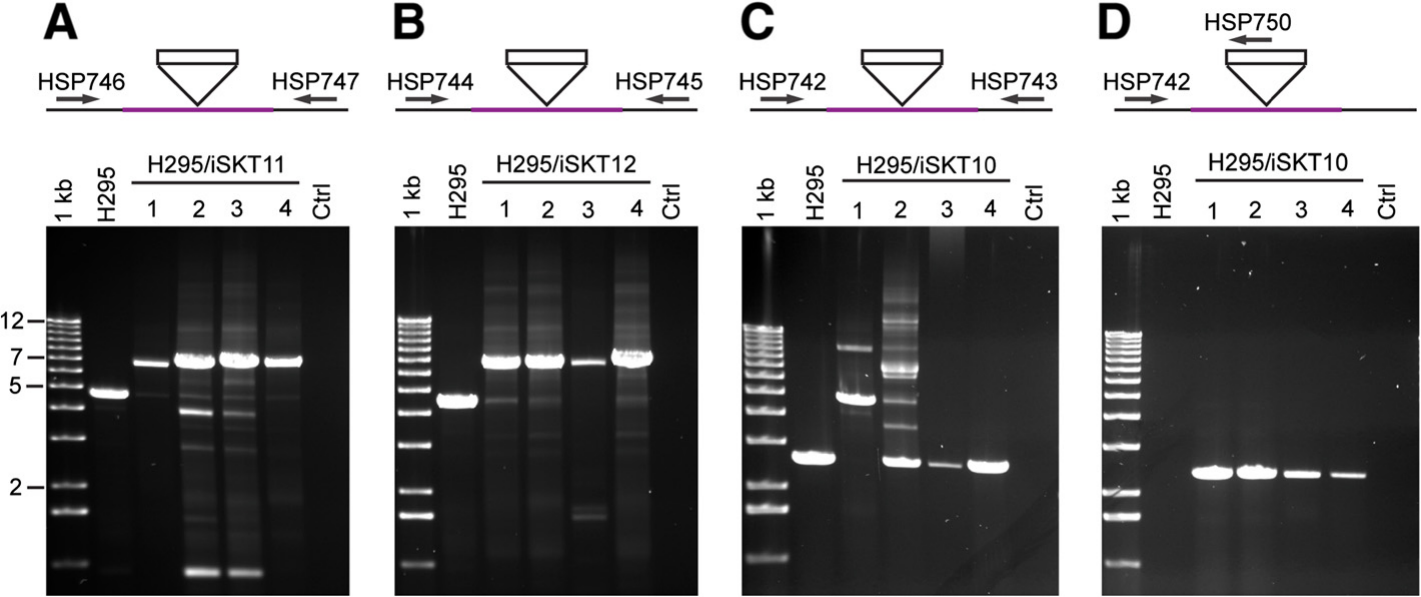
**PCR analysis of transposon insertions.** Linear fragments containing transposon TrpA-cat-Mu and flanking *H. volcanii* DNA were released from plasmid vectors by restriction digestion and transformed into H295 cells. Four clones from three transformations were isolated, their DNA was extracted, and PCR was used to detect the presence of the inserted transposon. **(A)** H295/iSKT11 clones were analyzed with primers HSP746 and HSP747. **(B)** H295/iSKT12 clones were analyzed with primers HSP744 and HSP745. **(C)** H295/iSKT10 clones were analyzed with primers HSP742 and HSP743 as well as **(D)** with primers HSP742 and HSP750. White rectangles indicate the 2.2 kb transposon. Flanking DNA from the integrated fragments is shown with purple. The primer recognition sites are shown with arrows. H295 indicates control PCR with DNA from the parental strain. Crtl indicates template-free control reactions. A 1 kb DNA ladder (Invitrogen) was used as a size standard.

PCR was then used to verify proper targeting. Initially, genomic DNA was isolated from four clones obtained from each of the three transformations (H295/iSKT10, H295/iSKT11, H295/iSKT12), and DNA was amplified from the corresponding loci (Figure 2). Properly targeted clones were expected to generate a PCR fragment with a 2.2 kb size increase (TrpA-cat-Mu transposon length) when compared to the corresponding fragment from the parental strain H295. Diagnosing successful targeting, the clones from transformations H295/iSKT11 and H295/iSKT12 produced prominent 6.9 kb and 6.7 kb fragments, respectively (Figure 2A, B). However, most clones also portrayed faint parental-size fragments, likely indicating the presence of a small number of non-mutated chromosome copies in the studied clonal cell populations. The results with clones from H295/iSKT10 transformation were less uniform (Figure 2C), as a prominent diagnostic 4.8 kb fragment was produced by one of the clones. The other three clones portrayed a major parental fragment of 2.6 kb, although a faint diagnostic fragment was also visible with two of them. Thus, it seemed that the distribution of mutated versus parental minichromosomes was not uniform among the clones studied. This finding was somewhat surprising, as all of these clones grew well without tryptophan, indicating the obligate presence of the *trpA* selection marker. We hypothesized that correctly targeted minichromosomes nevertheless were present in all of these clones studied, but with a very low copy number when compared with the copy number of the wild type pHV3. To test this hypothesis, we devised a PCR strategy that amplified DNA only from a properly targeted minichromosome (Figure 2D). All of the four clones studied exhibited a diagnostic 2.3 kb fragment, indicating proper targeting. Taken together, the data from these experiments showed that linear selection-marker-containing genomic fragments can be targeted in a single step into specific loci, not only in the main chromosome but also in the minichromosome. The results also showed that a mixture of mutated and non-mutated chromosomes was present in cells even after the selection regime applied, and this was particularly evident with the minichromosome pHV3. We suspect these findings directly reflect the multicopy nature of *H. volcanii* chromosomes and processes involved in the segregation and homogenotization of its genome.

### *H. volcanii* mutant libraries with genomic insertions

**Figure 3:**
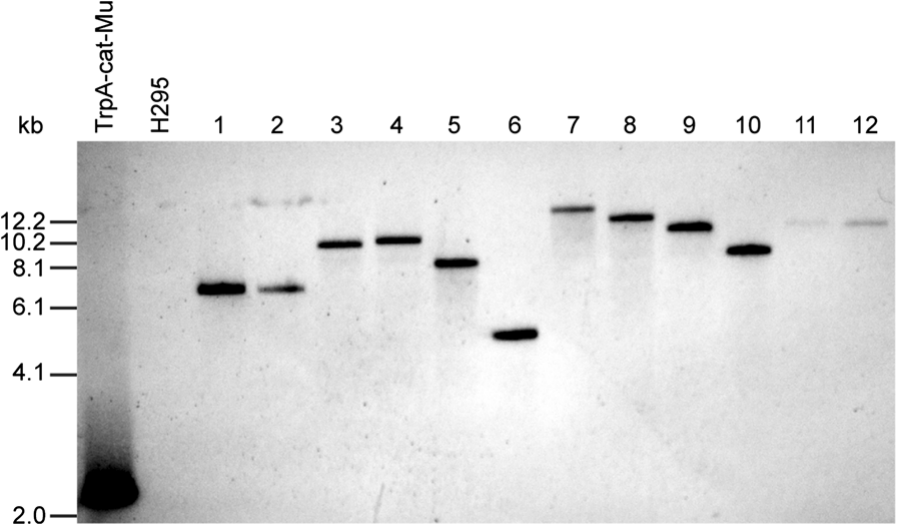
**Southern analysis of transposon insertion mutants.** Genomic DNA (2.5 μg) was digested with EcoRI + KpnI and probed with a transposon-specific probe. Purified TrpA-cat-Mu transposon (15.5 ng) and parental H295 DNA were used as positive and negative controls, respectively. Lanes 1 and 2 represent pigmentation mutants, lanes 3 and 4 auxotrophic mutants, and lanes 5 to 12 randomly selected uncharacterized mutants. Faint bands in lanes 11 and 12 are due to a smaller amount of DNA in these two samples.

To generate *H. volcanii* insertion mutants, a 4-6 kb fragment mixture was isolated from the plasmid library, and the mixture was used to transform H295 cells for chromosomal targeting *en masse*, in which each fragment would target its counterpart genomic locus (Figure 1B). In order to avoid a bias caused by restriction enzyme recognition site distribution, two enzyme pairs were used. Fragments released with XhoI + HindIII and KpnI + EcoRV double digestions both produced transformants with the efficiency of approximately 2 × 10^4^ CFU/μg DNA. For these two sets of fragments, we performed two transformations each, in total yielding approximately 4 × 10^4^ (XhoI + HindIII) and approximately 3 × 10^4^ (KpnI + EcoRV) colonies. To detect the presence and copy number of the transposon DNA, 12 of these clones were then studied by Southern hybridization using a transposon-specific probe (Figure 3). A single fragment was detected with all of the clones, indicating that at least the majority of the library clones harbored the transposon as a single copy. These data are fully consistent with proper targeting via homologous recombination.

### Pigmentation-defective mutants

*H. volcanii* synthesizes a distinct set of carotenoids [40], resulting in pink colonies and providing a convenient phenotype to validate a mutant library for gene discovery. Accordingly, we screened the above two libraries for mutants deficient in color formation (see Additional file 1). A total of 3.4 × 10^4^ colonies were visually inspected, and 18 whitish clones were discovered. To identify the genes affected, we determined by sequencing the transposon integration site in 15 of these clones. In each of them, the transposon was located in the *crtB* gene (HVO_2524). C*rtB* encodes phytoene synthase, which catalyzes the first specific step in the carotenoid synthesis pathway [41,42], logically explaining the observed phenotype and verifying that the library represents a potent tool for gene identification. Four different insertion sites in *crtB* were identified (Table 4), the number falling within the expectancy range of seven hits per gene on the average. The recovery of several identical sites was not surprising, as replicated sibling plasmids were necessarily present in the plasmid library prepared originally from 28,000 *E. coli* colonies.

**Table 4:** **Pigmentation-deficient mutants**

**Mutant**	**Integration site; orientation** ^**a**^	**Gene**	**Protein**
W-20	2,389,801; +	HVO_2524, *crtB*	Phytoene synthase
W-5, W-17	2,389,806; -	HVO_2524, *crtB*	Phytoene synthase
W-6, W-7, W-14	2,390,154; +	HVO_2524, *crtB*	Phytoene synthase
W-4, W-8, W-9, W-10, W-12, W-13, W-15, W-16, W-19	2,390,422; -	HVO_2524, *crtB*	Phytoene synthase

^a^The first change compared to *H. volcanii* chromosomal sequence (NC_013967.1) is indicated as the transposon integration site. The orientation is denoted as *trpA* direction compared to *H. volcanii* chromosome (5′ to 3′): +, same direction; -, opposite direction.

### Auxotrophic mutants

We screened a total of 4,100 mutants for auxotrophy by initially testing their ability to grow on Hv-Min minimal medium plates. Sixteen mutants did not grow, and their growth capacity was further studied on Hv-Min plates each supplemented with a single amino acid (Table 5). All biologically relevant amino acids were tested, and auxotrophic mutants were found for arginine (five clones), histidine (two clones), proline (two clones), tyrosine (one clone), isoleucine (one clone), and phenylalanine (one clone). Four of the sixteen mutants did not grow with any of these single amino acid supplements.

**Table 5:** **Amino acid auxotrophic mutants**

**Mutant**	**Auxotrophy**	**Integration site; orientation** ^**a**^	**Gene**	**Protein**
H295/6-78	Arginine	43,942; +	HVO_0044, *argB*	Acetylglutamate kinase
H295/6-100	Arginine	44,698: +	HVO_0045, *argC*	N-acetyl-gamma-glutamyl-phosphate reductase
H295/12-27	Arginine	44,905; -	HVO_0045, *argC*	N-acetyl-gamma-glutamyl-phosphate reductase
H295/10-36	Arginine	43,144; +	HVO_0043, *argD*	Acetylornithine aminotransferase
H295/17-48	Arginine	2,374,426; -	HVO_2508, *carA*	Carbamoyl-phosphate synthase small subunit
H295/13-54	Histidine	1,198,248; +	HVO_0431	HAD-superfamily hydrolase
H295/12-18	Histidine	1,198,248; +	HVO_0431	HAD-superfamily hydrolase
H295/21-77	Proline	786,914; +	HVO_0869	Glutamate synthase [NADPH] large chain
H295/24-65	Proline	1,051,586; -	HVO_1153	Hypothetical protein
H295/12-1	Tyrosine	1,198,243; +	HVO_1312	Prephenate dehydrogenase
H295/16-1	Isoleucine	578,651; -	HVO_0644, *leuA1*	2-isopropylmalate synthase/(R)-citramalate synthase
H295/24-63	Phenylalanine	399,114; -	HVO_0449, *pheA1*	Prephenate dehydratase
H295/9-8	Valine, Leucine, Isoleucine	1,374,516; -	HVO_1506, *ilvC*	Ketol-acid reductoisomerase
H295/4-26	Valine, Leucine, Isoleucine	1,375,827; +	HVO_1508, *ilvB1*	Acetolactate synthase large subunit
H295/12-44	Glycine, Serine, Threonine	2,805,618; +	HVO_2969, *thrC3*	Threonine synthase

^a^The first change compared to *H. volcanii* chromosomal sequence (NC_013967.1) is indicated as the transposon integration site. The orientation is denoted as *trpA* direction compared to *H. volcanii* chromosome (5′ to 3′): +, same direction; -, opposite direction.

The transposon insertion sites in the growth-defective mutants were then determined (Table 5). All the five arginine auxotrophs had the transposon inserted in the genes known to be involved in arginine biosynthesis: *argB*, *argC, argD*, and *carA,* of which *argC* was targeted at two different sites. The two histidine auxotrophs were identical with the transposon inserted in the same position in HVO_0431 (similarity to HAD-superfamily hydrolases). One proline auxotroph had the insertion in HVO_0869 (glutamate synthase subunit) and the other in HVO_1153 (hypothetical protein). In the tyrosine auxotroph the transposon interrupted HVO_1312 (prephenate dehydrogenase, a known enzyme on the tyrosine biosynthesis pathway). The isoleucine auxotroph harbored the transposon in *leuA1*, proposed to encode either 2-isopropylmalate synthase or highly similar (R)-citramalate synthase. In the phenylalanine auxotroph the transposon was inserted in *pheA1* (prephenate dehydratase, known to participate in phenylalanine biosynthesis). In summary, the transposon insertion sites pinpointed both genes known to be involved in amino acid biosyntesis and genes with no prior annotation.

Of the four mutants not growing with single amino acid supplements, three harbored the transposon in a gene involved in amino acid biosynthesis. Two of them, *ilvC* (ketol-acid reductoisomerase) and *ilvB1* (acetolactate subunit), are needed for valine, leucine, and isoleucine biosynthesis. Indeed, the addition of the mixture of these three supplements restored the growth of these mutants. The third identified gene, *thrC3* (threonine synthase) is required in glycine, serine, and threonine metabolism, and the addition of all three of these amino acids restored the mutant growth. The sequence data from the fourth growth-deficient mutant suggested that a larger scale genome rearrangement might have taken place, possibly due to an initial insertion of two transposons (see Discussion). As the revelation of the exact alteration would have required substantial extra efforts in sequencing, this mutant was not studied further.

## Discussion

More than 100 sequenced archaeal genomes are currently available, and the number is rapidly increasing (UCSC Archaeal Genome Browser [43-45]). Although the sequence data are critically important for gene discovery, such data cannot be used to predict archaeal gene or protein functions if homologous counterparts have not been characterized in other organisms. For example, only about 66% of the predicted genes in seven sequenced haloarchaeal genomes was assigned with a hypothetical protein function in a recent study [46], leaving a significant portion of the genomes non-annotated. As simple sequence comparisons cannot reveal genes behind unique archaeal features, experimental approaches are needed to advance our knowledge about the biology of these intriguing organisms. Various types of mutant libraries constitute an important resource for genetics/genomics studies, and those containing randomly distributed genomic insertions have been particularly useful for the identification of gene functions. Transposon-mediated mutagenesis provides a straightforward means to produce insertion libraries and has been widely applied in bacteria and eukaryotes [47-50]. However, the use of insertion mutagenesis has been very limited with archaea [20,21,23]. This is presumably due to the difficulties in methods development caused by the challenging biology of most archaeal species. Furthermore, inadequately characterized native transposon systems [51] and potential incompatibility problems with heterologous systems may have hindered progress towards the efficient utilization of transposition-based techniques in archaea.

The aim of this study was to develop a robust insertion mutagenesis methodology for *H. volcanii*. We chose to use minimal-component Mu *in vitro* transposition [33,52] for the distribution of insertions in DNA and homologous recombination machinery of *H. volcanii* for genomic integration [30-32]. Mu *in vitro* reaction yields transposition products highly efficiently and with a relatively low target site selectivity [33,53]. In practice, the distribution of integrations is essentially random along any longer segment of DNA. These characteristics have made the Mu reaction ideal for a variety of molecular biology [54-56], protein engineering [57-63], and genomics [27,64-67] applications. Also in this study, the reaction provided a straightforward means to deliver selection cassettes into random positions along the *H. volcanii* chromosomal DNA. In order to maximize the frequency of gene targeting via homologous recombination into the *H. volcanii* genome, we chose to use the strain H295, which is devoid of Mre11and Rad50 proteins. In the wild type strains these proteins form a complex restraining homologous recombination, and in the H295 mutant strain this activity is reported to be increased roughly 100-fold [37]. As the targeting frequency with H295 in our experiments was approximately 2 × 10^4^ CFU/μg DNA, it is likely that the generation of sizeable mutant libraries can also be accomplished with strains exhibiting the wild type level of homologous recombination activity.

In order to produce a high quality insertion mutant library for *H. volcanii*, we paid careful attention to the details of the strategy design and optimized several key steps in the techniques used. (1) Three different restriction endonucleases and partial digestions were used to fragment *H. volcanii* genomic DNA, effectively reducing the bias caused by restriction site distribution and guaranteeing an extensive coverage of the genome with overlapping fragments. (2) The size-selection following the *in vitro* transposition reaction prior to ligation ensured that 4-6 kb genomic inserts were enriched in the plasmid library. Accordingly, the effective elimination of short fragments guaranteed that practically all of the cloned inserts were of suitable length for gene targeting. (3) Our preliminary *en masse* targeting trials with circular plasmids resulted in a large fraction of *H. volcanii* clones that had not experienced genomic insertions but contained replicative plasmids harboring the selection cassette used and an *H. volcanii* origin of replication (data not shown). These plasmids evidently represented molecules, in which the transposon was originally integrated in the vicinity of an *H. volcanii* origin of replication, and ultimately resulted in shuttle plasmids capable of replication in both *E. coli* and *H. volcanii*. This prompted us to use linear DNA fragments for targeting and to eliminate even trace amounts of DNA circles from the targeting preparation. As alkaline plasmid preparation methods produce a fraction of collapsed supercoiled plasmid forms, which enter the cells effectively and cannot be digested with restriction endonucleases, we removed these plasmid forms by purifying the plasmid library using cesium chloride gradient centrifugation. In addition, the DNA to be transformed was size-selected prior to transformation using a preparative agarose gel, a technique which also selects against circular plasmid forms. We believe that the circle-elimination procedures were particularly pivotal for the successful generation of a high-quality insertion mutant library.

The plasmid library was made from 28,000 *E. coli* colonies. Theoretically, assuming random transposon insertion into *H. volcanii* DNA, the probability (*P*) at which every gene is targeted at least once can be calculated using the formula *P* = 1 - (1 - x/G)^n^ [68], where x is the average gene size, G is the genome size, and n is the number of mutants. The size of the *H. volcanii* genome is 4.01 Mb, and the number of genes is 4,130 [10]. Thus, the average gene size is 971 bp. With these numbers, the probability of every gene being tagged in the plasmid library was 0.9989, indicating that all of the *H. volcanii* genes were represented in this library with a very high likelihood. Note that these calculations do not take into account the known coding density of 86% [10]. It should be emphasized that the plasmid library generated contains a full repertoire of both essential and non-essential *H. volcanii* genes. However, the original diversity of the plasmid library could not be maintained in the final *H. volcanii* genomic insertion mutant library. This is because an insertion into any essential gene would result in cell death upon mutant genome homogenotization, leading to a situation where the cell would not be able to survive following the loss of wild type chromosome copies. Likewise, it should be remembered that not all of the insertions generate a loss-of-function mutation.

As dam-methylation had earlier been shown to reduce transformation efficiency in *H. volcanii* [69], we used both Dam^+^ and Dam^-^ DNA in our experiments. The transformation efficiency with plasmid DNA was shown to be dependent on its methylation status, being ten times higher with unmethylated than methylated DNA. This difference is smaller than the 100- or 1,000-fold difference described earlier for *H. volcanii* [69,70]. The reason for this apparent discrepancy is not known but may plausibly relate to the use of different *H. volcanii* strains. Intriguingly, the methylation status had no effect on the transformation efficiency with linear fragments. This is a novel finding, as (to the best of our knowledge) comparative transformation experiments with linear DNA fragments have not previously been conducted with *H. volcanii*. These data might indicate that targeting is a relatively rapid process and/or that the homologous recombination machinery of *H. volcanii* protects the incoming DNA from degradation. This conclusion is in line with earlier studies with *H. volcanii*, showing that integration into the chromosome can rescue methylated foreign DNA from being destroyed [70]. An important practical consequence of our findings is that the linear DNA used for transformation can be purified from a variety of sources, and it does not need to be passaged through a Dam^-^ microbial strain.

We performed important quality measures to control the library generation process. Initially, we studied the accuracy of the targeting procedure by introducing three separate linear transposon-tagged *H. volcanii* DNA fragments into cells. With each fragment, the transposon was found in the expected locus in the genome, confirming that targeting proceeded accurately via homologous recombination. Thus, linear tagged DNA fragments can be used effectively in a fast, one-step procedure to generate site-specific insertions into the *H. volcanii* genome. However, in theory there exists a possibility that the integration of incoming DNA could also follow a non-homologous recombination pathway. On the basis of our data, we believe that such pathways, if operational, do not nevertheless prevail in *H. volcanii*. This is demonstrated by our results from the gene discovery experiments: sixteen out of eighteen phenotypically screened insertion sites (in four pigmentation-defective and twelve auxotrophic mutants) identified a known gene on a logical biochemical pathway, a result incompatible with a dominating integration via non-homologous recombination machinery. As the second quality measure, we determined the number of transposon copies present in the genome of twelve insertion mutant library clones. In each case Southern analysis revealed only one resident copy. As the stoichiometry in the transformation of linear fragments should favor single-copy genomic integrations (only one out of approximately 2,000 cells becomes transformed, data not shown), the result was not surprising. Thus, each mutant clone should harbor only one tagged gene, establishing the genomic insertion library as a powerful tool for genetic screens.

**Figure 4:**
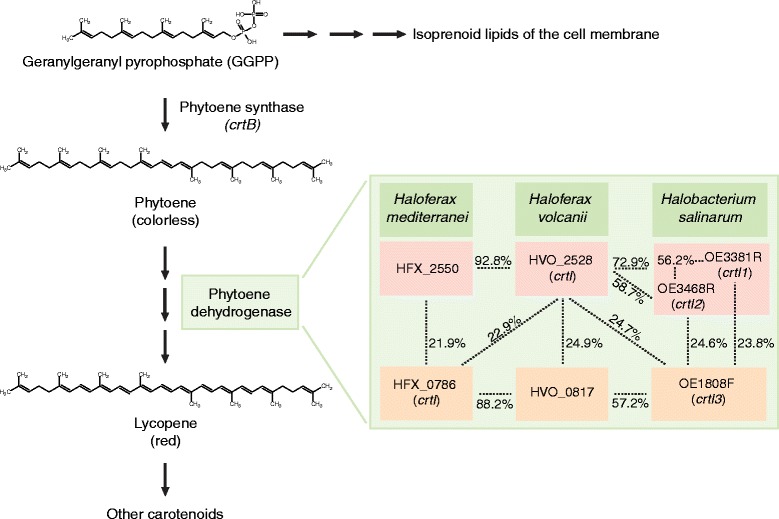
**Carotenoid biosyntehesis pathway in haloarchaea.** The pathway from geranylgeranyl pyrophosphate to lycopene is shown. The insert portrays the comparison of the deduced phytoene dehydrogenase sequences of *Haloferax volcanii* (NC_013967.1) to those of *Haloferax mediterranei* (NC_017941.2) and *Halobacterium salinarum* (NC_010364.1). The numbers indicate the percentage of identical amino acids between the compared proteins. Pairwise analyses were made by using EMBOSS Water [73].

To validate the insertion mutant library for gene identification, we initially searched for mutants defective in pigment formation. The *crtB* gene, identified in the search, encodes phytoene synthase (Figure 4). This enzyme, which has been characterized in several bacterial, archaeal, and plant species, catalyzes the first specific step in the carotenoid biosynthesis pathway, that is, the formation of phytoene from two molecules of geranylgeranyl pyrophosphate (GGPP) [41,42]. The loss of pigmentation was thus an expected outcome upon the interruption of *crtB*. We were not able to identify insertion mutations in the genes required for the synthesis of GGPP. This molecule is required not only for the carotenoid biosynthesis, but it is also a precursor of isoprenoid lipids, essential components of the archaeal cell membrane [71]. Thus, it is likely that insertions into any of the genes needed for GGPP biosynthesis would result in cell death, and our data are consistent with this supposition. In archaea, the colorless phytoene is converted into the red-colored lycopene by phytoene dehydrogenase [42,72]. In *H. volcanii*, the *crtI* gene (HVO_2528) has been annotated to encode this enzyme [10]. If this gene is solely responsible for the phytoene hydrogenase activity in *H. volcanii*, we should have pinpointed it with our screen. A closer look at the *H. volcanii* genome annotation revealed HVO_0817, annotated to encode a flavin-containing amine-oxidoreductase. This enzyme family contains various amine oxidases, also including phytoene dehydrogenases and related enzymes. Homology searches with other halophilic archaea indicated that the deduced protein sequence of HVO_0817 shares a high degree of similarity with *Haloferax mediterranei* and *Halobacterium salinarum* phytoene dehydrogenases: 88.2% and 57.2% identical amino acids with the products of *crtI* (HFX_0786) and *crtI3* (OE1808F), respectively (Figure 4). Thus, *H. volcanii* HVO_0817 most likely encodes a phytoene dehydrogenase. Considering that the theoretical likelihood for an average gene not being tagged in our study was as low as 0.0011, the fact that we were unable to pinpoint either HVO_2528 or HVO_0817 strongly suggests that these two genes encode overlapping functions and thus complement each other. As similar redundant phytoene dehydrogenase genes are present also in *H. mediterranei* and *H. salinarum* (Figure 4), such a gene arrangement may be a common feature among haloarchaea.

The screen for amino acid auxotrophic mutants mostly identified genes known to be involved in amino acid biosynthesis: *argB*, *argC*, *argD,* and *carA* for arginine, HVO_1312 (prephenate dehydrogenase) for tyrosine, *ilvC* and *ilvB1* for valine, leucine and isoleucine, and *thr3* for glycine, serine and threonine. Interestingly, the transposon insertion in the isoleucine auxotroph was in the *leuA1* gene, which has been proposed to encode either a 2-isopropylmalate synthase required for leucine biosynthesis or an (R)–citramalate synthase required for isoleucine biosynthesis [10]. Our results thus support the latter hypothesis. The proline auxotroph (H295/21-77) had the transposon inserted in HVO_0869, which encodes glutamate synthase (NADPH) large chain. Glutamate synthase is a key enzyme in ammonia assimilation, and the interruption of HVO_0869 is expected to result in unbalanced glutamate and nitrogen metabolism. More work would be needed to find out why the growth defect of this mutant is restored by the addition of proline, but not by any other amino acid. In addition to these known amino acid biosynthesis genes, we obtained two auxotrophic mutants having insertions in putative or unidentified genes: HVO_0431 (HAD-superfamily hydrolase) for histidine and HVO_1153 (hypothetical protein) for proline. Our study thus proposes the involvement of these genes in amino acid biosynthesis pathways, illustrating the huge potential of the library for the discovery of novel genes.

The copy number of the *H. volcanii* major chromosome is dependent on growth phase and conditions, being as high as 30, while the minichromosome copy numbers are somewhat lower (approximately 20) [11]. A study with *H. mediterranei* minichromosome pHM300 suggests that the replication of the major and minor chromosomes is controlled independently [74], and we suspect that this may be the case also in *H. volcanii.* In transposition mutagenesis, the transposon is integrated into the DNA of one chromosomal copy, while others remain wild type. Therefore, during the first few cell generations following mutagenesis, the transposon-containing genome copies are necessarily in a minority. As the mixture of mutated and non-mutated chromosomes may persist in a cell for some time, the cells thus being heterozygous, it may be intuitively somewhat surprising that loss-of function mutants could be isolated in our study relatively easily.

Our results suggest that chromosome homogenotization takes place under the selection pressure applied, and the PCR data on the targeting into the major chromosome supports this conclusion. The findings are consistent with the results of an earlier *H. volcanii* study, showing that genome copies will be equalized under selection pressure relatively rapidly [19]. In the paper the authors suggested efficient gene conversion machinery as a primary driving force behind the phenomenon, although mutually non-exclusive uneven segregation could not entirely be ruled out. Our pHV3 targeting data showed variation in chromosome homogenotization, possibly indicating that this process may be slow or inefficient with minichromosomes.

There are a few important issues that need to be considered when generating and using mutant libraries for microbes as described in this study: (1) Two copies of the transposon may be integrated *in vitro* into the same target DNA fragment, although rarely, as the reaction stoichiometry favors single insertions. If such a fragment passes the size selection applied, it may experience recombination between the transposon copies. This can occur either in *E. coli* or in the target organism, and it could ultimately lead to a deletion or inversion, both relatively uninformative with regard to gene discovery. (2) The mutagenesis strategy produces siblings in the plasmid library. Thus, following transformation into the target organism, the recovery of identical genomic insertion clones is expected, which was particularly well illustrated with our *crtB* mutants. Therefore, a large number of mutants with a desired phenotype should be collected in order to guarantee the finding of all possible insertion sites. (3) The target genome may contain regions that cannot be cloned into a plasmid vector, and such regions will not be recovered as transposon-tagged clones in the final insertion library. (4) The possibility of polar effects should be remembered when working with insertion mutant libraries of microbes. (5) Although rarely, spontaneous mutations may generate false positive results, in which case the transposon tag will identify a gene not involved in the observed phenotype. (6) The genetic screens should always be regarded as a first step towards gene discovery. In order to verify the genotype-phenotype connection, complementation or site-directed mutagenesis studies should be executed with each potential candidate gene.

## Conclusions

To conclude, we present here the generation of the first genome-wide insertion mutant library for haloarchaea using the model archaeon *H. volcanii*. The library includes clones that harbor a single transposon insertion in their genome, and it can be used for gene identification in *H. volcanii*. The strategy used provides a straightforward general means to generate comprehensive mutant libraries, is characteristically species non-specific and should readily be transferable to other archaeal species. Thus, the library represents a significant step towards advanced genetics studies in archaea.

## Methods

### Strains and culture conditions

Microbial strains and their use are described in Table 2. *E. coli* strains were cultured at 37°C in Luria-Bertani (LB) medium or on LB-agar plates [75] supplemented with ampicillin (Amp, 100 μg/ml) and/or chloramphenicol (Cam, 10 μg/ml) when required. For *H. volcanii*, cultured at 45°C, MGM (18%) was used as a rich medium, Hv-Ca as a medium to select for the Trp marker, and Hv-Min as a minimal medium [76]. For culture dishes, these media were solidified by the addition of Bacto agar (Difco, 15 g/L). In Hv-Min dishes most amino acids were used at a concentration of 50 μg/ml. Exceptions were histidine (at 65 μg/ml) and threonine (at 10 μg/ml). Uracil was used at 50 μg/ml.

### Enzymes, reagents, oligonucleotides, DNA, and molecular biology techniques

Restriction endonucleases, calf alkaline phosphatase and T4 DNA ligase were from New England Boston, MA (Ipswich, MA, USA) and used as recommended by the supplier. MuA transposase and Phusion DNA polymerase were from Finnzymes (currently Pittsburgh, PA, Waltham, MA, USA). Plasmid DNA isolation kits and RNase A were from Bethlehem, PA (Düren, Germany). Antibiotics, amino acids, and uracil were from Allentown, PA (St. Louis, MO, USA). Oligonucleotides used are described in Table 6 and plasmids in Table 1. Standard DNA manipulation methods were used [75]. *E. coli* competent cells were prepared and electrotransformed as described previously [27]. *H. volcanii* transformation was done using a published protocol [31]. *H. volcanii* chromosomal DNA was isolated at room temperature as follows: cells were collected by centrifugation from 3 ml of late exponential phase culture. A total of 200 μl of ST buffer (1 M NaCl, 20 mM Tris-Cl, pH 7.5) was added and the cells were resuspended, after which they were lysed by the addition of 200 μl of lysis solution (100 mM ethylenediaminetetraacetic acid (EDTA), pH 8,0, 0.2% SDS) [76]. DNA was extracted twice with 1 volume of phenol with one hour incubation at 60°C during the first extraction phase. Subsequently, DNA was extracted with 1.5 volume of chloroform and ethanol precipitated. Finally, DNA was dissolved in TE buffer (10 mM Tris-Cl, pH 7.5) containing RNase A (0.3 mg/ml). For the amplification of *H. volcanii* genomic DNA by Phusion DNA polymerase, PCR conditions recommended for GC-rich DNA were used (GC-buffer and 5% dimethyl sulfoxide (DMSO)).

**Table 6:** **Oligonucleotides used in the work**

**Oligonucleotide**	**Sequence (5′ to 3′)**	**Comment**
HSP675	GCGCGCGGATCCAGTTATGTGCGTTCCGGATG	Cloning of *Pfdx* + *trpA*
HSP683	GCGCGCGGATCCCGTGGATAAAACCCCTCGTT	Cloning of *Pfdx* + *trpA*
HSP734	CGTTGTAAAACGACGGCCAGTG	Sequencing from pBlueScript SK+, forward
HSP735	GGGAACAAAAGCTGGAGCTCC	Sequencing from pBlueScript SK+, reverse
HSP742	AACGCCTCGTAGAGCGTGTA	PCR spanning the insertion site of iSKT10. Forward, 5′ at 228,414 of pHV3^a^
HSP743	CGTCGTCGAACGTACCTCAT	PCR spanning the insertion site of iSKT10. Reverse, 5′ at 230,985 of pHV3^a^
HSP746	TGAGTCGAGACGGAGCGAGA	PCR spanning the insertion site of iSKT11. Forward, 5′ at 1,530,341 of *H. volcanii* chromosome^a^
HSP747	CCCACGAGAAAGGCGAGAAC	PCR spanning the insertion site of iSKT11. Reverse, 5′ at 1,534,771 of *H. volcanii* chromosome^a^
HSP744	GTTTCTTGGCGAGGGGTTC	PCR spanning the insertion site of iSKT12. Forward, 5′ at 385,560 of *H. volcanii* chromosome^a^
HSP745	CGAGACCTTCTCCAGCTCGT	PCR spanning the insertion site of iSKT12. Reverse, 5′ at 390,228 of *H. volcanii* chromosome^a^
HSP750	CCCCGTGGAGGTAATAATTGACG	Sequencing of TrpA-cat-Mu insertion site, PCR of iSKT10 insertion site
HSP751	CGTCGCAACGCCCACCGC	Sequencing of TrpA-cat-Mu insertion site

^a^Numbering according to *H. volcanii* D2 pHV3 (NC_013964.1) and chromosome (NC_013967.1), respectively.

### *E.coli*/*H.volcanii* shuttle transposon

TrpA-cat-Mu transposon is a derivative of Cat-Mu [33] with an added *H. volcanii*-specific selectable gene cassette (*trpA* gene driven by *Pfdx* promoter). It was constructed by initially amplifying a PCR fragment with the primer pair HSP683/HSP675 (Table 6) and plasmid pTA351 (Table 1) as a template. The fragment was trimmed with BamHI and ligated into a BamHI site of Cat-Mu such that the selectable marker genes (*trp* and *cat*) are transcribed in opposite directions (Figure 1A). TrpA-cat-Mu was maintained within its carrier plasmid pMPH20 (Table 1), from which it was isolated by BglII digestion and purified using anion exchange chromatography as described [33].

### Plasmid library of transposon-tagged *H. volcanii* DNA

*H. volcanii* strain H53 genomic DNA was digested partially with HpaII, AciI and TaqI in three separate reactions, purified by phenol extraction and ethanol precipitated. The DNA fragments were used as targets in three separate Mu *in vitro* transposition reactions [33] using TrpA-cat-Mu as a donor DNA. The reaction products were then pooled and purified as above. Anion exchange chromatography [33] was used to size-select 4 to 6 kb fragments, which were then ligated into pBlueScript SK+ cleaved with ClaI. The ligation mixture was electrotransformed into *E. coli* DH10B, and transposon-containing plasmid clones were selected on culture plates containing Amp and Cam. Approximately 28,000 colonies were pooled, and the cells were grown for 2.5 hours in liquid culture containing Amp and Cam. Plasmid DNA was isolated from the pool, yielding a primary plasmid library with *H.volcanii* genome segments tagged with transposon DNA as inserts. An aliquot from the library was electrotransformed into *E. coli* DH5α using the above selection, and approximately 600,000 colonies were pooled. The cells were grown and plasmid DNA was isolated as above. To eliminate non-supercoiled plasmid forms, the plasmid preparation was further purified using CsCl-gradient ultracentrifugation [75].

### *H. volcanii* insertion mutant library

Transposon-tagged *H. volcanii* DNA fragments were released from the plasmid library using XhoI + HindIII double digestion. Linear 4-6 kb fragments were isolated using preparative 1% agarose gel (Rockland, MD, Basel, Switzerland). Several 1 μg aliquots of linear DNA were used to transform [31] *H. volcanii* H295 cells. Following transformation, cells were collected by centrifugation and resuspended in 1 ml of transformant dilution solution [31], after which 335 μl of 80% glycerol – 6% salt water [76] was added. The cells were frozen in aliquots under liquid nitrogen and stored at -75°C. For the evaluation of transformation efficiency, one aliquot was thawed, the cells were plated on selective media and cultured for seven days. To avoid the potential diversity decrease caused by unfavorable restriction site distribution, another library was made by releasing the transposon-tagged *H. volcanii* DNA fragments using KpnI + EcoRV double digestion.

### Southern analysis

EcoRI + KpnI double-digested chromosomal DNA (2.5 μg) was separated on a 0.8% SeaKem LE agarose gel (Lonza). The DNA was transferred onto Hybond-N+ -membrane (Pittsburgh, PA/GE Healthcare, Pittsburgh, PA, USA). Southern hybridization was performed as described [75] with biotin-labeled (Biotin DecaLabel DNA Labeling Kit, Thermo Scientific) *cat-*gene-specific DNA (PvuII-ScaI fragment of TrpA-cat-Mu). Biotin Chromogenic Detection Kit (Thermo Scientific) was used for visualization.

### Mutant isolation

An aliquot of *H. volcanii* H295 insertion mutant library was plated on selective media and cultured for seven days. Colonies were visually inspected for pigmentation deficiency, and white normal-sized colonies were isolated. To isolate auxotrophic mutants, 4,100 mutant clones were streaked onto Hv-Ca and Hv-Min plates. Mutants failing to grow on Hv-Min were isolated and studied for their ability to grow on supplemented Hv-Min plates containing one or several amino acids.

### Determination of transposon location

Initially, chromosomal DNA from isolated mutant clones was cleaved with KpnI, EcoRI or SphI (not cleaving the transposon DNA) in separate reactions. From each reaction, the ensuing fragments were cloned into the corresponding site of pUC19 (Table 1). Plasmid clones containing transposon-tagged *H. volcanii* genomic DNA fragments were selected on LB-plates containing Amp and Cam. Plasmid DNA was isolated and the sequence from each transposon border was determined using transposon-specific primers HSP750 and HSP751 (Table 6). DNA sequence determination was performed at the sequencing facility of the Turku Centre for Biotechnology by using 3130xl Genetic Analyzer (Pittsburgh, PA Waltham, MA, USA). Genomic transposon insertion site loci were identified by using NCBI blastn suite [77] and the publicly available genome sequence of *H. volcanii* D2 strain [10]. Functions of enzymes along the carotenoid and amino acid biosynthesis pathways were assessed by using KEGG Pathway Maps [78].

## Additional file

Additional file 1: Figure S1. Pigmentation-deficient mutants. Carotenoid biosynthesis mutants and the control strain H295/pTA231 were cultured on a Hv-Ca dish at 45°C for four days and subsequently incubated at room temperature for four days. One representative mutant for each identified *crtB* insertion site is shown. Colonies were imaged using Olympus SZX12 microscope and Jenoptic ProgRes SpeedXT core 5 camera.

## Abbreviations

Amp: ampicillin
Bp: base pairs
Cam: chloramphenicol
CFU: colony-forming unit
EMS: ethylmethanesulfonate
Kb: kilo-base pairs
LB: Luria-Bertani
PCR: polymerase chain reaction
