# Supplementary figures and images for: Generation of comprehensive transposon insertion mutant library for the model archaeon, Haloferax volcanii, and its use for gene discovery
Source: BMC Biol. 2014 Dec 9;12:103. doi: 10.1186/s12915-014-0103-3 (PMC4300041; doi:10.1186/s12915-014-0103-3)

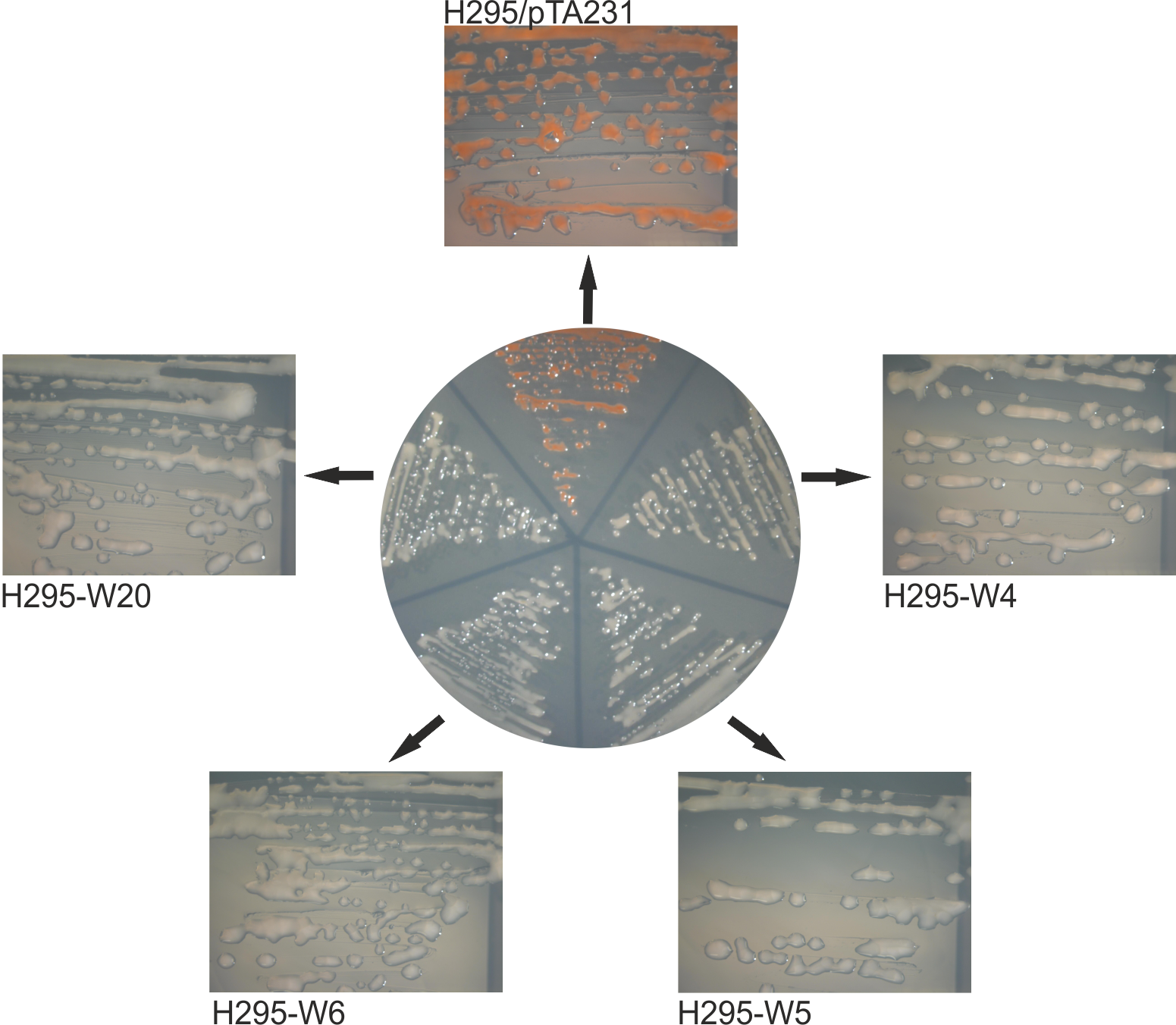

Supplement: Additional file 1: Figure S1. — Pigmentation-deficient mutants. Carotenoid biosynthesis mutants and the control strain H295/pTA231 were cultured on a Hv-Ca dish at 45°C for four days and subsequently incubated at room temperature for four days. One representative mutant for each identified crtB insertion site is shown. Colonies were imaged using Olympus SZX12 microscope and Jenoptic ProgRes SpeedXT core 5 camera. [file 12915_2014_103_MOESM1_ESM.tiff]
